# Death in Soothing Syrup

**Published:** 1890-04

**Authors:** 


### Death in Soothing Syrup.

INFANTS KILLED BY BEING GIVEN A PATENT NOSTRUM.

QUEBEC, November 28.—Coroner Belleau, in a letter to the Provincial Health Board yesterday, directed the attention of the authorities to a patent medicine called "soothing syrup," alleging that it was a deadly poison and had already caused the death of half a dozen children.

The case which directed Dr. Belleau's attention to the poison was that of an infant which was taken ill on Saturday night. On Sunday the father went to a drug store bought a bottle of the soothing syrup and administered a dose to the child. It immediately went to sleep and remained so until Monday morning, when it died.

The coroner found that the patent medicine contained nearly 40 per cent. of liquid extract of opium. He also learned that five other families had lost a child each in the same way, in all, six cases.

The syrup has been secured by the coroner. The directions, order a teaspoonful every hour until the child becomes quiet. Dr. Belleau says two tablespoonfuls would kill any ordinary man.—*Special to the Examiner.*

